# Probiotic consumption reduces alveolar bone loss and kidney damage in pregnant rats with experimental periodontitis

**DOI:** 10.1002/jper.11389

**Published:** 2025-09-26

**Authors:** Átila V. V. Nobre, Pedro H. F. Silva, Marina C. G. Del‐Arco, Raquel F. Gerlach, Rene S. Oliezer, José E. Tanus‐Santos, Luciene C. Figueiredo, Janaina S. A. M. Evangelista, Flávia A. C. Furlaneto, Michel R. Messora, Sérgio Luiz Salvador

**Affiliations:** ^1^ Department of Oral and Maxillofacial Surgery and Periodontology Ribeirao Preto Dental School, University of Sao Paulo Ribeirao Preto Sao Paulo Brazil; ^2^ Department of Clinical Analyses Toxicology and Food Science, School of Pharmaceutical Sciences of Ribeirao Preto, University of Sao Paulo Ribeirao Preto Sao Paulo Brazil; ^3^ Department of Basic and Oral Biology Dental School of Ribeirao Preto, University of Sao Paulo Ribeirao Preto Sao Paulo Brazil; ^4^ Department of Pharmacology Ribeirao Preto Medical School, University of Sao Paulo Ribeirao Preto Sao Paulo Brazil; ^5^ Department of Periodontology Dental Research Division, Guarulhos University Guarulhos Sao Paulo Brazil; ^6^ Faculty of Veterinary Medicine State University of Ceara Fortaleza Ceara Brazil

**Keywords:** periodontitis, *Porphyromonas gingivalis*, pregnancy, probiotics

## Abstract

**Background:**

*Bifidobacterium animalis* subsp. *lactis* HN019 (*B. lactis* HN019) is a probiotic bacterial strain with immunomodulatory properties. Its benefits have been observed in healthy and systemically compromised animals with periodontitis (PD). Our objective was to investigate the local and systemic effects of the systemic administration of *B. lactis* in pregnant rats with experimental periodontitis (EP).

**Methods:**

For this, 48 pregnant rats were divided into four different groups (*n* = 12/group): Control (C), Probiotic (PROB), Periodontitis (PD), and Periodontitis + Probiotic (PD‐PROB). EP was induced using a mixed model of cotton ligature placement and oral gavage of *Porphyromonas gingivalis* W83. On gestational day 19, the animals were euthanized for sample collection and analysis. Jaws, kidneys, and urine samples were collected for microtomographic, histological, histomorphometric, and biochemical analyses. The data were statistically analyzed (*p *< 0.05) using nonparametric tests (Kruskal–Wallis) and analysis of variance (ANOVA) followed by Tukey and Dunn post hoc tests.

**Results:**

EP resulted in local and systemic damage, such as alveolar bone loss (ABL) and kidney damage, and the consumption of *B. lactis* HN019 resulted in improvements in these parameters. Regarding mandibular analyses, the PD‐PROB group showed greater bone volume in the furcation region, a greater number and thickness of bone trabeculae, and less bone porosity and separation between trabeculae compared to the PD group (*p *< 0.05). Regarding kidney analysis, the PD‐PROB group showed lower glomerular and Bowman's capsule diameters and circumferences compared to the PD group (*p* < 0.05).

**Conclusion:**

Probiotic consumption reduced damage in mandibular bone and kidney tissues in pregnant rats with EP.

**Plain language summary:**

Periodontitis (PD) is a destructive periodontal disease that can lead to tooth loss. The treatment for PD consists of scaling and root planing to remove calculus and plaque deposits; however, some systemic conditions make it difficult to control this disease. Probiotic bacteria have emerged as adjuvants in the treatment of infectious diseases, and their benefits have been demonstrated in the management of PD. The aims of the present study were to evaluate whether PD has a negative impact on pregnancy and whether the probiotic strain *Bifidobacterium animalis* subsp. *lactis* HN019 can reduce this impact. For this purpose, 48 pregnant rats were divided into four experimental groups (Control, Probiotic, PD, and PD + Probiotic), and samples of maternal and pup weights, as well as placentas, mandibles, urine, and kidneys, were collected and analyzed. We observed that PD negatively impacted pregnant rats, resulting in greater alveolar bone loss, increased expression of proteinuria and creatinine in urine, and kidney damage; systemic probiotic administration reduced these harmful effects. In addition, pups, as well as mothers supplemented with probiotic, exhibited higher weights and larger litter sizes, suggesting a beneficial effect on nutrition and development during pregnancy.

## 1 INTRODUCTION

Periodontitis (PD) is a chronic inflammatory condition associated with an alteration of the natural balance of the subgingival microbiota. This alteration favors the overgrowth of certain gram‐negative bacterial species considered periodontopathogens that are involved in oral dysbiosis.^1^ The knowledge about the effects of PD on systemic health has given rise to the term periodontal medicine.^2^ This area of periodontics studies the relationship between periodontal diseases and systemic diseases, including diabetes mellitus (DM),^3^ cardiovascular diseases,^4^ and adverse pregnancy outcomes (APO).^5^

The relationship between PD and APO has been widely explored in the literature. Premature birth (PB), low birth weight (LBW), and preeclampsia (PE) are the main gestational complications investigated for a possible association with PD through different mechanisms of action.^6^ The etiology of APO is multifactorial, but it is known that maternal infection and inflammation directly affect neonatal outcomes.^9^ Pro‐inflammatory mediators secreted by inflammatory cells present in periodontal pockets can disseminate through blood vessels to other tissues and organs.^6^, ^7^ Thus, the plausibility of the association between APO and PD lies in the principle that inflammatory mediators produced and disseminated by the periodontal region can activate the inflammatory cascades that initiate parturition.^8^ Furthermore, there is evidence of the metastatic infection of periodontopathogens and their toxic byproducts into the systemic circulation, reaching the fetoplacental unit.^6^, ^7^, ^8^ Thus, advances in science regarding the pathophysiology of APO indicate the prevention of infections and maternal metabolic disorders as strategies for eradication.^5^, ^6^

The gold standard treatment for PD consists of mechanical debridement of the biofilm and subgingival calculus through scaling and root planing (SRP) of the surfaces affected by PD, associated with effective control of the supragingival biofilm.^9^ However, this therapy alone is not always able to reverse the clinical situation, especially in diseases in advanced stages and degrees. The use of probiotic bacteria emerges as an adjuvant therapeutic approach to SRP. The Food and Agriculture Organization of the United Nations and the World Health Organization describe probiotics as “live microorganisms that, when administered in adequate amounts, confer a health benefit on the host.”^10^ The mechanisms of action of probiotics are attributed to their immunomodulatory capacity and their direct interaction with the immune system.^11^ In addition to the immunomodulatory effect, probiotics show microbiological benefits through the inhibition of the growth and survival of other bacterial species by direct killing or competitive exclusion. The secretion of growth inhibitory factors, reduction of fitness through alteration of microbial signaling, and occupation of available niches are mechanisms of action of probiotic strains.^12^

Several studies have described the potential benefits of probiotics in systemic health, such as control of total cholesterol and low‐density lipoprotein levels, regulation of the renin‐angiotensin‐aldosterone system, and management of medical disorders, such as gastrointestinal diseases, metabolic syndrome, and irritable bowel syndrome.^13^, ^14^, ^15^ As for the use of probiotics in pregnancy, it has been reported that dietary intervention with *Lactobacillus rhamnosus* and *Bifidobacterium animalis* subsp. *lactis* (*B. lactis*) reduced the incidence of gestational diabetes in humans.^16^ Clinical and preclinical studies have shown therapeutic benefits of probiotics in the prevention of preterm labor through modulation of systemic and intrauterine inflammation and by modulating the maternal vaginal microbiota.^17^, ^18^

Regarding the possible association between APO and PD and the impact of the probiotic, an observational study comparing women who had PB with women who had normal pregnancies reported that women who had PB exhibited worse periodontal parameters and higher expression of pro‐inflammatory cytokines (such as interleukin [IL]‐1β, IL‐6, IL‐8, tumor necrosis factor [TNF]‐α, and prostaglandin [PG]2) in the gingival crevicular fluid (GCF) and serum compared with women with normal pregnancies.^19^ A case–control study comparing women who had APO (PB or LBW) with women who had normal pregnancies showed that APO was associated with worse periodontal parameters and greater expression of placental inflammatory markers.^20^ Probiotic consumption as an adjunct to SRP demonstrated a significant reduction in IL‐1β and IL‐8 levels and an increase in IL‐10 levels, an anti‐inflammatory cytokine, in GCF in patients with PD.^11^ Thus, according to the principle of systemic dissemination of inflammatory mediators from the periodontal region, it is believed that probiotic supplementation, through immunomodulation of the periodontal inflammatory process and the microbiological benefits presented, may have a positive impact on APO.

The relationship between APO and PD remains controversial in the literature, and to date, there is no consensus on a causal relationship. Observational studies point to a plausible possibility. In addition to the multifactorial etiology and confounding factors of both APO and experimental periodontitis (EP), methodological variability in clinical studies contributes to inconclusive findings. Methodological drawbacks include, but are not limited to, the different approaches used to assess PD, variations in study design, sample size, and population heterogeneity.^19^, ^20^, ^21^ Only one study reported the impact of probiotic therapy on gingivitis associated with pregnancy.^22^ Despite the growing interest in this area, no previous studies have investigated the impact of probiotic therapy on the association between PD and pregnancy. Thus, the objectives of the present study are to evaluate the effects of PD during pregnancy in rats and to determine if probiotic therapy with *B. lactis* HN019 can promote benefits in this interface.

## 2 MATERIALS AND METHODS

### 2.1 Animals and sample size calculation

This study was carried out in accordance with the ethical principles of animal experimentation and following the ARRIVE (Animal Research: Reporting of In Vivo Experiments) guidelines.^23^ All procedures were reviewed and approved by the Ethics Committee on Animal Experimentation at the School of Dentistry of Ribeirao Preto—University of Sao Paulo (protocol 2018.1.800.58.8).

The calculation of the sample size was performed according to a previous study.^24^ Considering that some animals could not develop PD due to the loss of the ligature and the possibility of some rats not becoming pregnant, four animals per group were added as a safety margin, totaling 12 animals per group.

### 2.2 Experimental design and groups

**FIGURE 1:**
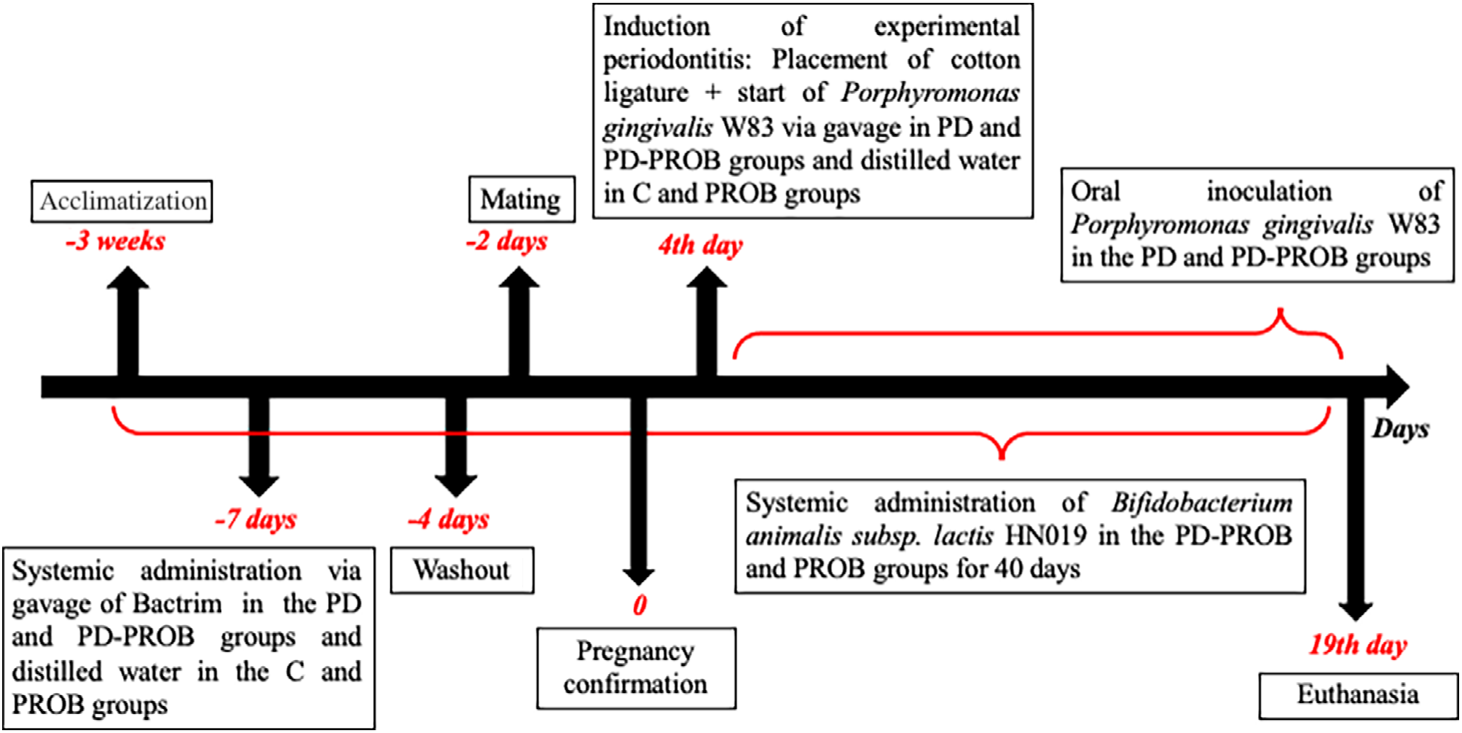
Experimental design. C, Control; PD, Periodontitis; PD‐PROB, Periodontitis + Probiotic; PROB, Probiotic.

Forty‐eight female and 24 male Sprague–Dawley rats of the SPF strain (free of specific pathogens) were used from the Central Animal Facility at USP Ribeirao Preto, aged between 7 and 8 weeks. The room was acclimatized to a temperature of 23 ± 2°C and maintained on a 12/12‐h light–dark cycle. They were fed a selected solid diet and had access to water ad libitum. The mating protocol was implemented when all females reached 10 weeks of age, in a dark environment, using the harem system, which involves two females for one male.^25^ For confirmation of pregnancy, vaginal lavage was performed to observe the sperm using optical microscopy. The experimental design is depicted in Figure 1.

Female pregnant rats were randomly allocated into one of the following groups (*n* = 12/group): C (Control, without PD), PROB (Control + Probiotic), PD (induced PD), and PD‐PROB (induced PD + Probiotic).

### 2.3 PD induction

PD was induced according to a previous study,^26^ using a combination of two techniques: cotton ligature placement and oral gavage of *Porphyromonas gingivalis* W83 (Pg) to ensure both local periodontal and systemic inflammatory effects. The combination of two PD induction methods seeks to mimic what happens in the oral cavity of humans with PD: the presence of a plaque‐retentive factor (ligature) and bacteremia present in daily events, such as chewing and swallowing (Pg gavage). The PD induction protocol began with the systemic administration of Bactrim*—sulfamethoxazole 200 mg and trimethoprim 40 mg for 7 days (once a day) for indigenous microbiota depletion, followed by 3 days of washout. Each tablet was macerated, diluted in 1 mL of distilled water, and inoculated via gavage in the animals that received the infection protocol. Animals that did not receive the infection protocol (C and PROB groups) received a gavage of 1 mL of distilled water as a sham procedure.

### 2.4 Ligature

At gestational day 4, the animals were anesthetized with an intraperitoneal injection of xylazine (10 mg/kg of body weight) and ketamine (80 mg/kg of body weight). A cotton ligature was placed around the mandibular first molars (MFM) of the animals, as previously described.^24^

### 2.5 Pg inoculation

Oral gavage of Pg in the PD and PD‐PROB groups started on gestational day 4 and continued for 15 days, as previously described.^26^ The animals that did not receive the infection received 2% carboxymethylcellulose via gavage on the same days and at the same times.

### 2.6 Preparation of probiotic cultures and administration to animals

*B. lactis* HN019 was prepared and cultured according to the protocol described previously.^24^ The quantitative standardization of the inocula was obtained by determining the optical density (OD) in a spectrophotometer at a wavelength of 625 nm, as well as by counting, in duplicate, the number of colony‐forming units (CFU)/mL. Thus, an OD of 2.898 corresponded to the standardized inoculum with 1.0 × 10^11^ CFU/mL. In the PROB and PD‐PROB groups, the probiotic was added daily to the animals' water in the proportion of 1 × 10^11^ CFU from the time of their arrival at the animal facility until the end of the experiment, with a mean daily consumption of 47.5 mL per animal.

### 2.7 Euthanasia and sample collection

At gestational day 19, the animals were weighed and anesthetized by inhalation of 2% isoflurane, and euthanasia was performed by administering an overdose of xylazine and ketamine. An incision was made in the abdominal region to remove the pups, placentas, and kidneys. Pups and placentas were carefully removed from the uterus and washed with ice‐cold (4°C) 0.9% saline. Urine was collected immediately after euthanasia, directly from the bladder for biochemical analysis. The right hemimandibles were used for microtomographic analysis and the left jaws for histopathological analysis.

### 2.8 Maternal, fetal, and placental parameters

Maternal weight (MW), pups’ weight (PW), number of pups per mother (PN), and placental weight were analyzed and measured using a high‐precision scale. Length and weight were expressed in centimeters (cm) and grams (g), respectively.

### 2.9 Histological processing and histopathological analysis of periodontal tissues

To confirm PD and evaluate whether the probiotic has any beneficial effect on periodontal tissues, the ligature region underwent histopathological analysis. After routine laboratory processing,^27^ two sections representing the most central buccolingual portion of the furcation area of the left MFM were stained using the hematoxylin and eosin (H&E) technique and selected for histopathological analysis. With the aid of light microscopy, the histopathological conditions of the periodontal tissues in the furcation region of the first lower molar were analyzed, considering the content of the periodontal inflammatory infiltrate, extent of inflammation, connective tissue pattern, and profile of the alveolar bone present.

### 2.10 Microcomputed tomography analysis of mandibles

To confirm PD and evaluate whether the probiotic has any beneficial effect on bone tissue, microcomputed tomography (micro‐CT) of the jaws was performed. This analysis evaluated alveolar bone loss (ABL) and bone profile in the furcation region of the MFM. For this, non‐demineralized specimens were scanned by a cone‐beam micro‐CT system (Skyscan 1174).† The X‐ray generator was operated with an acceleration potential of 60 kV, a current of 165 µA, and an exposure time of 650 ms per projection. Images were produced with a voxel size of 6 × 6 × 6 µm.

Appropriate software‡ was used to generate three‐dimensional models, which were then rotated into a standard position according to the following criteria: (1) In the transaxial plane, the right MFM had its axis vertically positioned; (2) in the coronal plane, the mandibular bone was vertically oriented, with the mesial root of the MFM in the upper part of the image; and (3) in the sagittal plane, the occlusal surface of the MFM was horizontally positioned. Linear measurements of alveolar bone levels were performed at four different sites: buccal, lingual, interproximal, and at the furcation. For buccal and lingual sites, on the transaxial image passing through the distal root of the MFM, the linear distances from the cementoenamel junction (CEJ) to the buccal/lingual alveolar bone crest (ABC) were measured. For interproximal sites, the coronal dataset was analyzed using appropriate software.§ The distance between the last image showing the ABC, between the mandibular second molar (MSM) and MFM, and the first image showing the CEJ of MFM was measured. For the furcation site, on the sagittal image passing through both the mesial and distal roots of the MFM, the alveolar bone level was assessed by measuring the distance between the roof of the furcation and the ABC in the furcation area. The sum of the four linear measurements obtained from each animal was expressed as the alveolar bone level value. For volumetric measurements, a volume of interest (VOI‐prismatic section) was outlined from the apexes of all roots of MFM up to the roof of the furcation of MFM, touching the root surfaces in all images of the coronal dataset, using the same software (CT‐Analyzer) applied for the analysis of the interproximal site. The images were binarized so that bone and dental structures could be distinguished according to differences in density, using greyscale (inferior limit 65, superior limit 255; greyscale threshold 0‐255). This pattern of binarization was used for all samples. The following parameters were analyzed: (1) bone volume/total volume (BV/VT) in the area of interest (VOI), (2) bone porosity (BP), (3) trabecular thickness (Tb.Th), (4) trabecular number (Tb.N), and (5) trabecular separation (Tb.Sp). All micro‐CT analyses were performed as previously described^28^ and conducted by a blinded and calibrated examiner (P.H.F.S.).

### 2.11 Biochemical analyses

Urine was evaluated for the presence of biomarkers of PE and kidney injury, such as protein and creatinine (CR) expression. For this, after euthanasia, urine was collected directly from the bladder and stored in sterilized tubes. A random sample was used to quantify proteins and CR in the urine. The colorimetric test was employed, following the manufacturer's recommendations.‖ The red pyrogallol method was used to quantify proteinuria (PR), and the modified Jaffe's method was used for urine CR. The results of PR and CR were expressed in milligrams per deciliter (mg/dL).

### 2.12 Histopathology and histomorphometry analyses of kidneys

In addition to PR and urinary CR, renal structure was evaluated because of the multisystem involvement associated with APO. For this, the sagittal half of the right kidney was fixed in a 10% buffered solution and processed as described.^29^ Serial sections 5 µm thick were stained with H&E. Histomorphometric evaluation was performed according to a previous study^30^ by a blinded examiner (P.H.F.S.) using Image J software. The parameters assessed included the diameter and area of the glomerulus, the circumference of the glomerulus, and Bowman's capsule (µm^2^). Histopathological analysis was performed by a specialist in veterinary histology (J.S.A.M.E.), who was blinded to the experimental groups, and the evaluated parameters included the degree of inflammation, cellularity pattern, intratubular inflammatory infiltrate content, and renal tissue structure.

### 2.13 Statistical analyses

Normality and homoscedasticity of the data were checked using GraphPad StatMate Prism 9.0 software. The significance level was set at 5%. The significance of differences among groups for microtomographic, histomorphometric, and biochemical parameters was assessed by analysis of variance (ANOVA) followed by a post hoc Tukey test or Kruskal–Wallis test followed by a post hoc Dunn test.

## 3 RESULTS

All animals completed the experimental protocol; however, one animal from the PD group was excluded from the sample due to the loss of ligature. Statistical power was not affected.

### 3.1 Maternal, pup, and placental measurements

**FIGURE 2:**
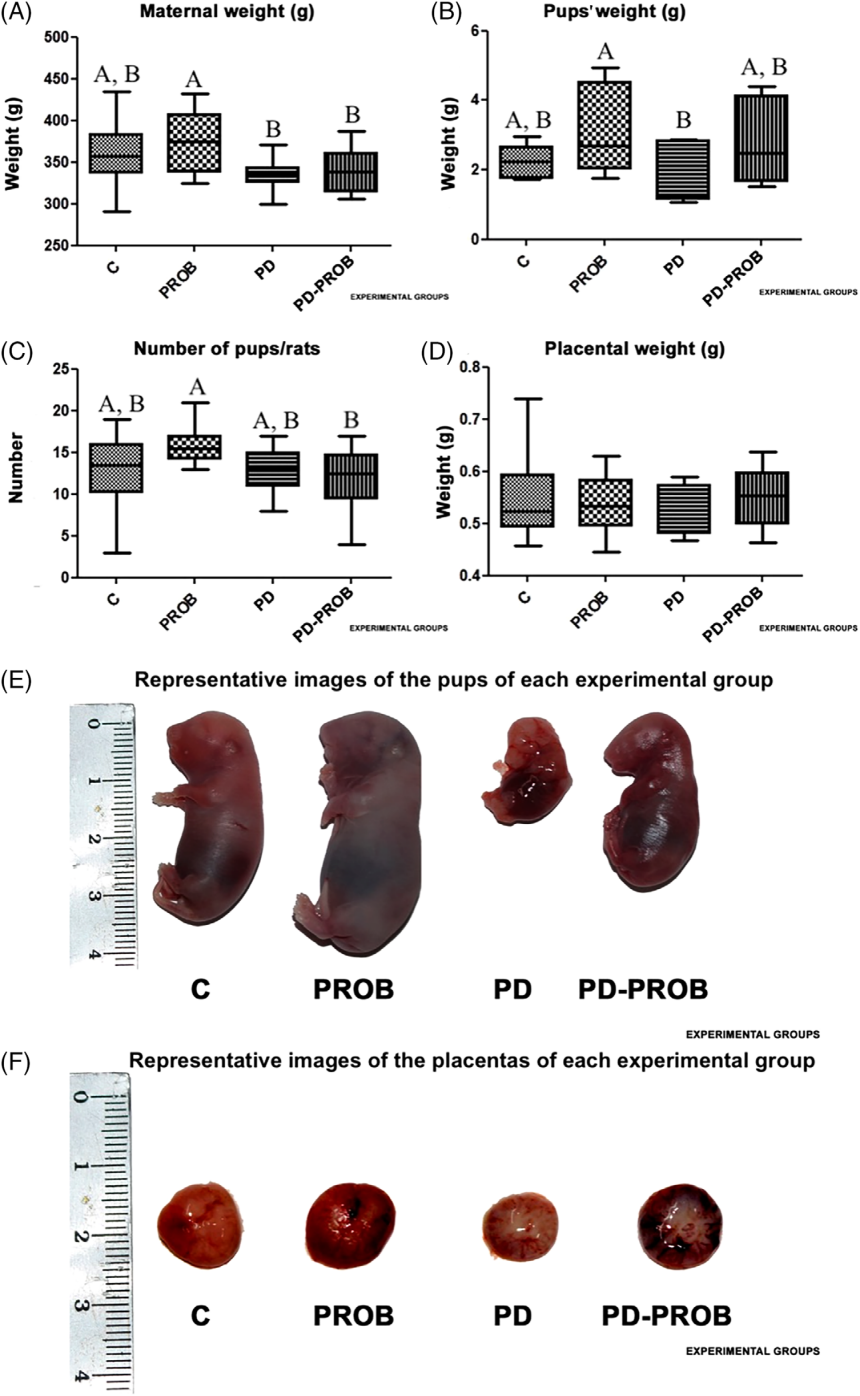
Anatomical parameters. (A) Maternal weight (MW) before euthanasia. (B) Pups’ weight (PW). (C) Number of pups per rat (PN). (D) Placental weight. (E) Representative images of pups from each experimental group. (F) Representative images of placentas from each experimental group. Number of samples per group: C (*n* = 12), PROB (*n* = 12), PD (*n* = 11), PD‐PROB (*n* = 12); MW: analysis of variance (ANOVA), post hoc Tukey test; PW: ANOVA, post hoc Dunn test; PN: ANOVA, post hoc Tukey test; placental weight: ANOVA, post hoc Tukey test. Different letters indicate statistical difference between groups (*p *< 0.05). C, Control; PD, Periodontitis; PD‐PROB, Periodontitis + Probiotic; PROB, Probiotic.

Figure 2 shows the medians and interquartile deviations for MW (Figure 2A), pups' weight (Figure 2B), PN (Figure 2C), and placental weight (Figure 2D). The number of samples for the outcomes MW, PW, PN, and placental weight per group was group C (*n* = 12), group PROB (*n* = 12), group PD (*n* = 11), and group PD‐PROB (*n* = 12). The PD group showed a significant reduction in MW compared with the PROB group (*p *< 0.05). Regarding the pups, the PD group had pups with a weight 40% lower compared to the PROB group (*p *< 0.05). The PROB group showed a 19% higher PN compared to the PD‐PROB group (*p *< 0.05). There was no statistical difference in placental weight between groups; however, there was a tendency toward lower values for the PD group. Figure 2E and F depicts representative images of the size of the pups and placentas among the groups.

### 3.2 Histopathological analysis of periodontal tissues

The number of samples for the histopathological results of periodontal tissue was group C (*n* = 12), group PROB (*n* = 12), group PD (*n* = 11), and group PD‐PROB (*n* = 12). Groups C and PROB presented periodontal ligaments within normal standards. Organized collagen fibers, numerous fibroblasts, blood vessels, and a discrete inflammatory infiltrate could be observed. The bone tissue in the furcation region presented many osteocytes and a regular ridge with apposition of osteoblasts (see Figure S1A–D in the online *Journal of Periodontology*). The PD group exhibited structural damage to the periodontal ligament. Disorganized and disconnected collagen fibers could be observed, along with the presence of interstitial edema and inflammatory infiltrate. In some regions, the connective tissue presented intense inflammatory infiltrate and the presence of osteoclasts (see Figure S1E,F in the online *Journal of Periodontology*). The PD‐PROB group also exhibited structural alterations in the periodontal ligament. When compared to the PD group, the connective tissue showed a greater quantity of fibroblasts, more organized collagen fibers interposed between the alveolar bone and the root cementum, and the presence of osteoclasts (see Figure S1G,H in the online *Journal of Periodontology*).

### 3.3 Micro‐CT analysis of mandibles

**FIGURE 3:**
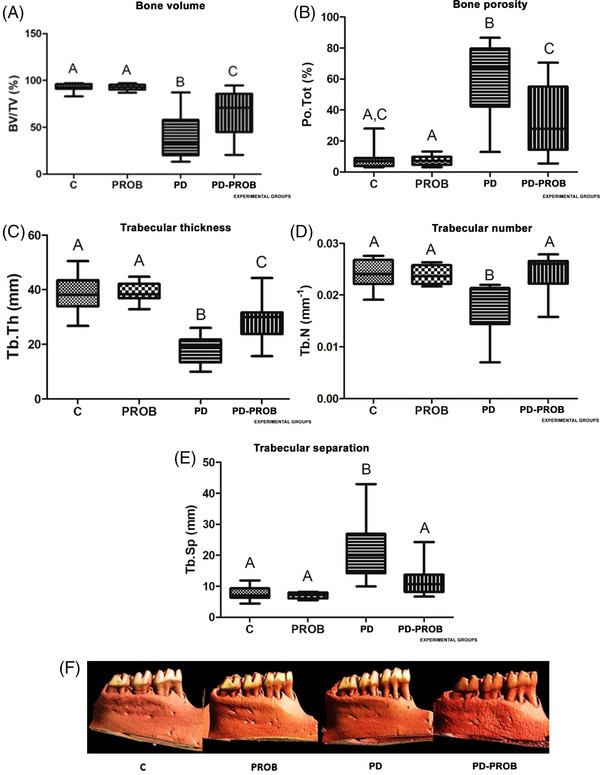
Microtomographic parameters. (A) Bone volume/total volume (BV/TV). (B) Bone porosity (Po.Tot). (C) Trabecular thickness (Tb.Th). (D) Trabecular number (Tb.N). (E) Trabecular separation (Tb.Sp). (F) Three‐dimensional rendered reconstructions of microtomographic sections of hemimandible of animals in each group. Number of samples per group: C (*n* = 8), PROB (*n* = 8), PD (*n* = 8), PD‐PROB (*n* = 8); BV/TV: analysis of variance (ANOVA), post hoc Tukey test; Po.Tot: ANOVA, post hoc Dunn test; Tb.N: ANOVA, post hoc Tukey test; Tb.Sp: ANOVA, post hoc Tukey test. Different letters indicate statistical difference between groups (*p *< 0.05). C, Control; PD, Periodontitis; PD‐PROB, Periodontitis + Probiotic; PROB, Probiotic.

The number of samples for micro‐CT results was *n* = 8/group. In the micro‐CT analysis, the PD group showed greater alveolar bone destruction compared with the PD‐PROB and other groups (*p *< 0.05). In the PD group, BV/VT reductions of 60% compared to the C and PROB groups and 20% compared to the PD‐PROB group were observed (Figure 3A). Regarding Tb.Th, the PD group showed a reduction of 50% compared to the C and PROB groups and 35% compared to the PD‐PROB group (Figure 3C). Regarding Tb.N, the PD group showed 20% fewer bone trabeculae (Figure 3D) in the VOI compared to the other groups (*p *< 0.05). Furthermore, BP was observed to be six times greater in the PD group compared to the C and PROB groups and two times greater in relation to the PD‐PROB group (Figure 3B). Regarding the thickness between trabeculae (Tb.Sp), the PD group showed values three times greater in relation to the C and PROB groups and two times greater in relation to the PD‐PROB group (Figure 3E; *p *< 0.05). Representative images of the three‐dimensionally rendered reconstructions of the microtomographic sections of the hemimandible of the animals in all groups can be seen in Figure 3F.

### 3.4 Biochemical analyses

**FIGURE 4:**
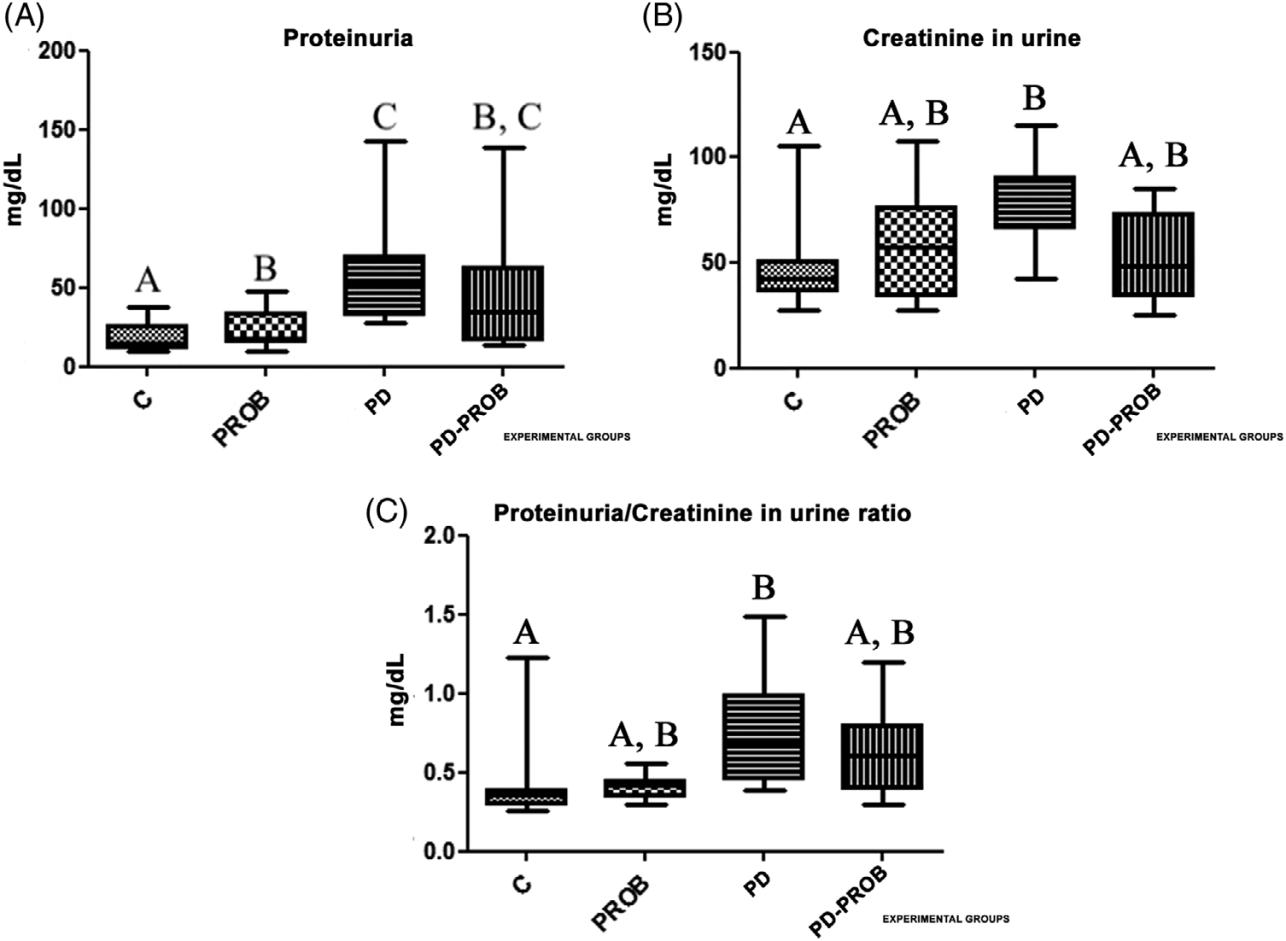
Biochemical parameters. (A) Proteinuria. (B) Creatinine in urine. (C) Urine proteinuria/creatinine ratio. Number of samples per group for proteinuria outcomes: C (*n* = 12), PROB (*n* = 12), PD (*n* = 10), PD‐PROB (*n* = 11). Sample sizes for creatinine and proteinuria/creatinine ratio in urine per group: C (*n* = 12), PROB (*n* = 12), PD (*n* = 10), PD‐PROB (*n* = 10). Different letters indicate statistical difference between groups (analysis of variance [ANOVA], post hoc Dunn test, *p *< 0.05). C, Control; PD, Periodontitis; PD‐PROB, Periodontitis + Probiotic; PROB, Probiotic.

The number of samples for the PR outcomes was as follows: group C (*n* = 12), PROB group (*n* = 12), PD group (*n* = 10), and PD‐PROB group (*n* = 11). For CR and the PR/CR ratio in urine, the numbers were: group C (*n* = 12), PROB group (*n* = 12), PD group (*n* = 10), and PD‐PROB group (*n* = 10). The reduced number of samples was due to the absence of the ideal amount of urine in the bladder of some animals needed to perform the biochemical reaction. Urinary protein excretion was three times higher in the PD group compared to the C group and two times higher compared to the PROB group (*p *< 0.05; Figure 4A). In addition, there was a statistically significant increase in urine CR excretion in the PD group compared with the C group (Figure 4B). The PD group showed a statistically significant increase in the urine PR/CR ratio compared to the C group (*p *< 0.05; Figure 4C).

### 3.5 Histopathology and histomorphometry analyses of kidneys

**FIGURE 5:**
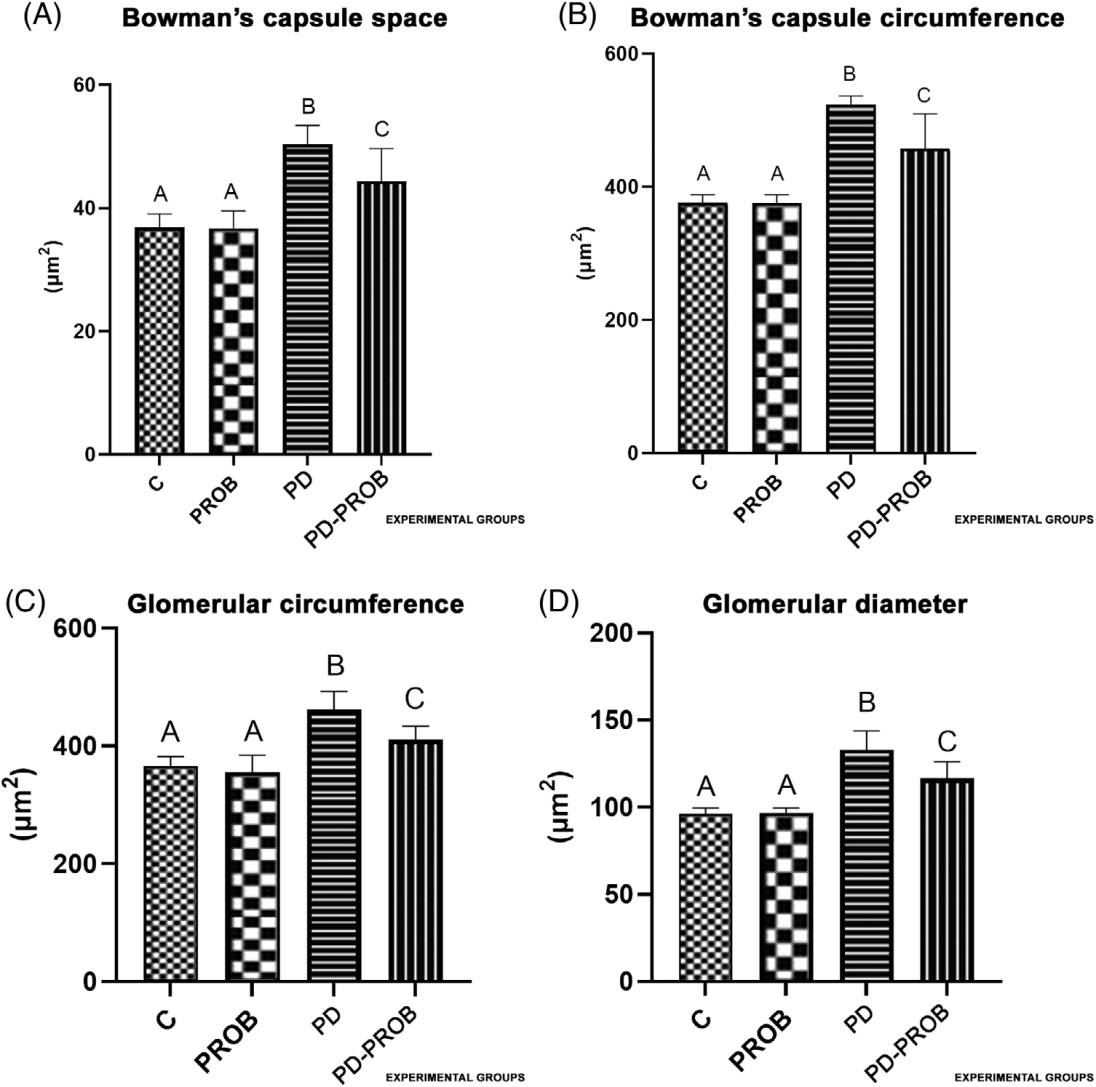
Kidney histomorphometric parameters. (A) Bowman's capsule space. (B) Circumference of Bowman's capsule. (C) Glomerular circumference. (D) Glomerular diameter. Number of samples per group for kidney outcomes: C (*n* = 8), PROB (*n* = 8), PD (*n* = 8), PD‐PROB (*n* = 8). Different letters indicate statistical difference between groups (analysis of variance [ANOVA], post hoc Dunn test, *p *< 0.05). C, Control; PD, Periodontitis; PD‐PROB, Periodontitis + Probiotic; PROB, Probiotic.

The number of samples for kidney outcomes was *n* = 8/group. The PD group presented a significant increase in Bowman's capsule space (Figure 5A), Bowman's capsule circumference (Figure 5B), glomerular circumference (Figure 5C), and glomerular diameter (Figure 5D) when compared with all the other groups (*p *< 0.05).

**FIGURE 6:**
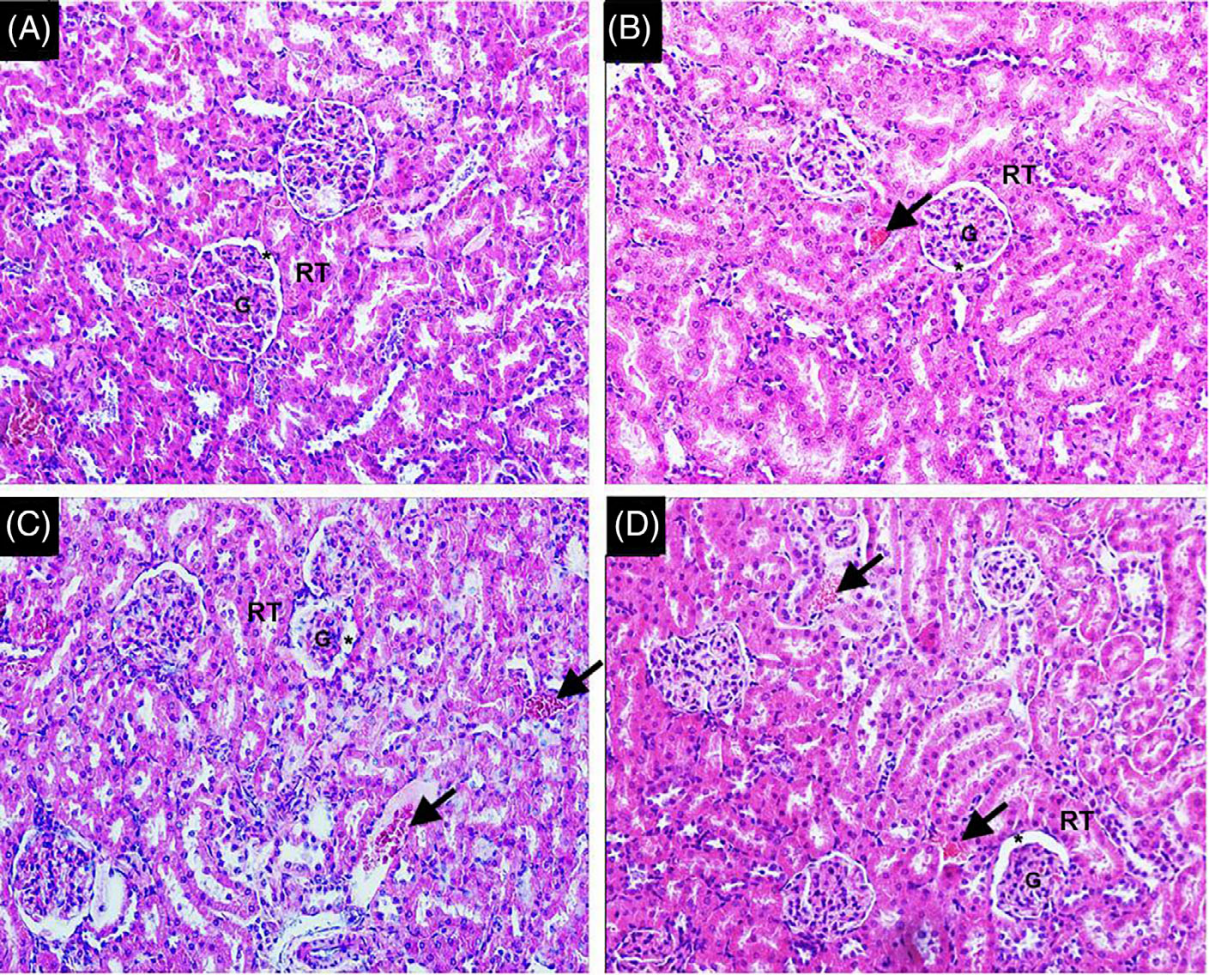
Representative photomicrographs of sagittal section of kidney. (A) C group. (B) PROB group. (C) PD group. (D) PD‐PROB group. *Indicates Bowman's capsule space; black arrow indicates congested vessels. Staining: hematoxylin & eosin; scale bar: 100 µm = magnification ×200. C, Control; G, Glomerulus; PD, Periodontitis; PD‐PROB, Periodontitis + Probiotic; PROB, Probiotic; RT, Renal Tubules.

In the histopathological analysis, the PD group exhibited alterations in the kidney tissue architecture, such as the presence of intratubular proteinaceous material, intravascular basophilic material, intense presence of congested vessels, slight hemorrhage, and moderate interstitial edema. Moderate to intense lymphoplasmacytic inflammatory foci in the marrow and mild fibrosis in the medullary region were also observed. The PD‐PROB group also presented signs of kidney damage but to a lesser extent when compared with the PD group (Figure 6A–D).

## 4 DISCUSSION

The present study is the first to evaluate the effects of probiotic therapy with *B. lactis* HN019 in pregnant rats with EP. According to our findings, a negative impact of PD on pregnant rats was observed at both local and systemic levels, including damage to fetal development, and probiotic therapy promoted beneficial effects. Among the negative effects of PD, we highlight ABL, an increase in the expression of PR and CR in the urine, kidney alterations, as well as reductions in maternal and pups' weight. Systemic administration of *B. lactis* HN019 reduced ABL in pregnant rats with EP and reduced damage in the kidneys. Despite the results, it is important to emphasize that the route, frequency, and dosage of probiotic administration, as well as the strain used, can impact the outcomes.

Regarding the anatomical parameters, it was observed that PD affected MW during pregnancy. The PD and PD‐PROB groups showed a significant weight reduction compared to the PROB group. In addition, the PD group had pups with lower birth weights compared to the PROB group. The PD‐PROB group had a smaller litter size compared to the PROB group. This finding is aligned with the study by Liang et al.,^31^ who reported a significant reduction in MW and PW and the number of pups in pregnant rats infected with *P. gingivalis* ATCC 33277 associated with ligature, and with previous findings from our research group.^26^ We can observe that pups from probiotic‐supplemented dams were significantly heavier. This can be explained by two potential reasons. First, it has been demonstrated that different strains of *Bifidobacterium* can induce maternal metabolic changes, such as increased gut acetate levels. This short‐chain fatty acid produced by gut bacteria, when overexpressed, induces effects by interacting with the maternal intestinal mucosa and regulates maternal gestational weight, neonatal length, and body weight and exerts protection against allergic airway disease in the developing pups.^32^ In addition, acetate crosses the placenta and can be used for cell metabolism, growth, and fetal‐placental function.^33^ Second, *Bifidobacterium breve* UCC2003 (*B. breve*) supplementation has been observed to reduce the barrier thickness of the mouse placenta, which facilitates the exchange of nutrients and gases between mother and pups. Barrier thickness is regulated by the placental‐specific transcript of IGF‐II (Igf2P0),^34^ and an increase in its expression has been demonstrated in mice supplemented with *B. breve*, which could explain, in part, the improvement in fetal weight associated with probiotic administration.^32^, ^33^, ^34^, ^35^

Placental weight was assessed, and the PD group tended to have lower values (0.530 ± 0.04) than the other groups (C = 0.549 ± 0.07; PROB = 0.538 ± 0.05; PD‐PROB = 0.549 ± 0.05), but without statistically significant differences (*p *< 0.08). The role of the placenta is to transport oxygen and nutrients from the mother to the fetus, and as pregnancy progresses, placental tissues undergo a series of structural and functional changes to ensure fetal health.^6^ Placental weight is closely related to the increase in surface area for the vascular exchange of nutrients, the lateral expansion of the chorionic disc, and the increase in placental thickness with arborization of the chorionic villi.^36^ Despite the interest in better understanding the role of the placenta in fetal development, the importance of placental weight is still not well explored.^37^ Some observational studies have developed standard percentile placental weight curves,^37^ stratified by major maternal comorbidities.^38^ To our knowledge, there are no studies that have performed this placental weight curve in animal models or that have evaluated placental weight in rats with PD. Placental weights have been reported to be significantly lower in mothers with hypertensive disorders, and placental weights have been found to be significantly higher in mothers with diabetic disorders. Obstetricians believe that placentas associated with fetal growth restriction (FGR) are significantly smaller than normal placentas. Fetal growth is dependent on placental growth.^37^ The association between lower placental weight and hypertension may suggest PE. We have previously demonstrated features of periodontitis‐induced PE.^26^ This finding, coupled with the LBW of the PD group pups, reflects the damage caused by periodontal infection.

The PD group had the worst results in micro‐CT analysis of the alveolar bone. Lower values of BV/VT and Tb.Th in the furcation region could be observed in the PD and PD‐PROB groups compared to the C and PROB groups. Lower Tb.N and higher Tb.Sp could be observed in the PD group compared to the other groups. A higher percentage of BP was observed in the PD and PD‐PROB groups compared to the PROB group. The PD‐PROB group showed twice the BP compared to the C and PROB groups. In contrast, the PD group exhibited six times higher BP compared to the same groups. This pattern could also be seen in relation to the lower BV/VT in the area of interest and in the Tb.Th, indicating a protective effect of the probiotic on ABL. These findings, as well as what was observed in the histopathological analysis of the furcation region, are in line with previous studies that used the *B. lactis* HN019 strain in systemically healthy or compromised rats.^24^, ^27^, ^28^, ^39^ *B. lactis* HN019 has immunomodulatory potential, with the ability to reduce Th1 pro‐inflammatory cytokines and increase the expression of Th2 anti‐inflammatory cytokines.^39^ Probiotic therapy, through the modulation of the intestinal microbiota, can promote benefits in suppressing ABL through different mechanisms of action.^40^ Among them, the modulation of the RANK–RANKL pathway and osteoprotegerin (RANK–RANKL–OPG). Probiotic bacteria can reduce bone resorption by increasing OPG expression.^41^

Normal pregnancy is characterized by adaptations in almost all maternal physiological systems. An example of these adaptations is the significant increase in plasma volume, which is essential for the development, oxygenation, nutrition, and maturation of a new organ, the placenta.^6^, ^8^ The kidneys play a crucial role in maintaining homeostasis and blood pressure control, and pregnancy is the only situation in which plasma volume expansion and electrolyte retention are essential for the adaptation of the placenta and the developing pups.^42^ Failures in plasma volume expansion and renal adaptations through renal hemodynamics, morphology, and nephron transport can lead to FGR and hypertensive disorders of pregnancy such as PE. PR has traditionally been a marker of PE, but it is also a nonspecific indicator of kidney disease and may result from increased plasma protein concentration, increased glomerular permeability, and renal hemodynamic changes.^43^ Thus, we investigated the urinary biochemical profile, maternal renal histological and histomorphometric changes, and the impact of the probiotic. The PD group had significantly higher levels of PR and urine CR compared to the C group. Our findings are aligned with those of Mata et al. (2021),^26^ who reported a 5‐fold increase in PR levels in pregnant rats with PE, simulating an experimental PE model. Pregnant women with PD have been reported to have higher 24‐h PR values and an increased risk of PE.^44^ The random urine PR/CR ratio is an alternative, quick, and simple method of detecting and estimating the quantitative assessment of PR that eliminates the need for daily 24‐h urine collection in the monitoring of kidney disease, DM, hypertension, and PE.^45^ The efficacy of the urinary RP/RC ratio as a simple laboratory tool for predicting gestational disorders has been reported.^46^ The present study showed significantly higher levels, in mg/dL, of the PR/CR ratio in the urine of the PD group compared to the C group.

As for histological and histomorphometric parameters, we observed maternal renal alterations, and the systemic administration of *B. lactis* HN019 reduced these alterations. The PD group showed a significant increase in all parameters evaluated (space and circumference of Bowman's capsule and glomerular diameter and circumference) compared to the other groups. The PD‐PROB group showed lower values compared to the PD group and higher values compared to the control groups (C and PROB). Our results are in agreement with the results of França et al. (2017),^29^ who showed alterations in the kidneys of rats with ligature‐induced PD. It has been reported that ligature‐induced PD significantly reduced the number of glomeruli, negatively affected the renal corpuscle structure, and resulted in increased tubulointerstitial fibrosis. These adverse effects were amplified in the presence of nephrectomy (Nx)‐induced chronic kidney disease (CKD) in rats with PD compared to rats without PD.^47^ In addition, increased macrophage infiltration and renal TNF‐α expression were observed in rats with PD and CKD. The authors suggest that PD not only induces morphological changes in the normal kidney but also exacerbates morphological changes in the presence of CKD and that Nx‐induced glomerular reduction makes the kidney more sensitive to a secondary challenge, such as PD.^47^ Our findings showed renal histopathological changes as early‐stage damage, justified by the fact that the intensity and duration of the injury were not sufficient to cause significant alterations in the microscopic architecture of the kidney. In our experimental model, ligature was maintained for 14 days, whereas in the study by Lee et al. (2024), the ligature was maintained for 4 weeks. This may explain the more expressive renal morphological changes, although they did not contribute to the impairment of renal function in either study.

In this context, it was observed that patients with CKD have high levels of PD and that successful periodontal treatment can reduce the levels of systemic inflammation in patients with CKD.^48^ Regarding the effects of probiotics, a literature review with meta‐analysis of randomized controlled trials that evaluated the effects of prebiotics, probiotics, and synbiotics in patients with CKD is noteworthy. The most commonly used probiotics were *Bifidobacterium* spp. and *Lactobacillus* spp., and it was concluded that microbial therapies promoted significantly beneficial effects on systemic inflammatory markers (C‐reactive protein), indicators of oxidative stress (malondialdehyde, glutathione, and total antioxidant capacity), and lipid profile (total cholesterol, triglycerides, low‐density lipoprotein cholesterol, and high‐density lipoprotein cholesterol) in patients with CKD.^49^

Regarding the effects of probiotics on systemic health, it is important to emphasize that these depend on several factors, such as the strain used, dosage, duration of therapy, form of administration, and the host's microbiome. The host's intestinal microbiome, for example, under normal homeostatic conditions, performs functions ranging from preparing the innate and adaptive immune response to driving metabolism, producing vitamins, regulating hormone levels, and promoting neurological health.^50^ In addition to being complex, the intestinal microbiome is dynamic, responsive to external stimuli, and influenced by factors such as lifestyle, diet, genetics, and epigenetics.^12^ It has been observed that intestinal dysbiosis has a negative impact on systemic health, with repercussions on the central nervous system, and probiotics can act positively in regulating the intestinal ecosystem. Probiotics have been reported as potential therapies in dysfunctions of the microbiota–intestine–brain axis in different sexes and periods of life,^51^ in chronic diseases such as DM,^14^ arterial hypertension,^15^ in pregnancy,^16^, ^17^, ^18^ and in the management of PD.^11^, ^12^, ^24^, ^27^, ^28^, ^39^ The challenges for preclinical studies on probiotic intervention range from animal models to the complexity of the host microbiome while considering the factors mentioned above. The most commonly used animal model is rodents, but nonhuman primates and pigs can also be included. Nonhuman primates are valuable for studying probiotic intervention in metabolic syndrome, for example, since metabolic defects in monkeys tend to occur late due to overfeeding and are similar to those that occur in humans. Thus, experimental results can be better inferred for humans.^52^ Pigs, on the other hand, are large and expensive animals to manage. Another important point in the variables of the study with probiotics is the delivery vehicles. Commercially, dairy products predominate in the development of probiotic foods. Our research group reported that the use of milk as a delivery vehicle for the *B. lactis* HN019 strain potentiated the effects of this strain in rats with PD.^53^

This is a proof‐of‐principle study, with limitations inherent to animal studies, in which only one dosage and duration of probiotics were evaluated, and initial analyses were performed. For possible clinical application, additional studies need to be conducted, preferably in larger animals that are higher on the phylogenetic scale, including local and systemic microbiological and immunological evaluations, both in placental and periodontal tissues, to evaluate the impact of probiotic therapy in pregnant women with PD. In any case, if the findings of this proof‐of‐principle study are confirmed, the use of probiotics during the gestational period may become an important adjuvant tool for pregnant women with PD (alone or associated with other systemic diseases, such as diabetes, metabolic syndrome, or obesity) and may even help to limit the extent of periodontal therapy during pregnancy while avoiding systemic adverse effects resulting from the disease. Furthermore, a longer follow‐up time (e.g., longitudinal monitoring of pups), species‐specific responses, and the absence of dose–response tests or strain comparisons constitute future perspectives that may contribute to the knowledge in this area. Despite these limitations, it appears that probiotics have promising potential as an adjuvant strategy in the association between PD and pregnancy.

## 5 CONCLUSIONS

We can conclude that EP in pregnant rats causes local and systemic damage and that therapy with *B. lactis* HN019 reduces damage to mandibular and renal bone tissue in pregnant rats with PD.

## AUTHOR CONTRIBUTIONS

All authors have made substantial contributions to the conception and design of the study. Nobre A.V.V., Salvador S.L., Messora M.R., Furlaneto F.A.C., and Gerlach R.F. have been involved in the conception and/or design of the work. Nobre A.V.V., Silva P.H.F., Del‐Arco M.C.G., and Seabra R.S. have been involved in data collection and data analysis. Tanus‐Santos J.E., Figueiredo L.C., and Evangelista J.S.A.M. have been involved in data interpretation and drafting the manuscript. Salvador S.L., Nobre A.V.V., and Messora M.R. have revised the manuscript critically and have given final approval of the version to be published.

## CONFLICT OF INTEREST STATEMENT

The authors have no conflicts of interest to report.

## Supporting information



Supporting Information

Supporting Information

## Data Availability

The data that support the findings of this study are available upon request from the corresponding author. The data are not publicly available due to privacy or ethical restrictions.

## Footnotes

* Roche; Roche Chemicals and Pharmaceuticals Inc., Rio de Janeiro, RJ, Brazil.

† Bruker, Kontich, Belgium.

‡ Data Viewer version 1.5.0; Bruker, Kontich, Belgium.

§ CT‐Analyzer version 1.13.5.1+; Bruker, Kontich, Belgium.

‖ Gold Analisa, Belo Horizonte, MG, Brazil.
