# Supplementary material for: Probiotic consumption reduces alveolar bone loss and kidney damage in pregnant rats with experimental periodontitis
Source: J Periodontol. 2025 Sep 26;97(4):732–46. doi: 10.1002/jper.11389 (PMC13169480; doi:10.1002/jper.11389)
Supplement: Supplementary file 1 — Supporting Information [file JPER-97-732-s001.docx]

**Figure s1** – Histopathological evaluation of periodontal tissues in the furcation region of groups C (A, B), PROB (C, D), PD (E, F) and PD-PROB (G, H). The number of right hemi-mandible samples was as follows: group C (n=8), group PROB (n=8), group PD (n=8), group PD-PROB (n=8). Staining: hematoxylin & eosin. Scale bar: 200 µm = magnification ×100; scale bar: 100 µm = magnification ×200). FR = furcation roof; AB = alveolar bone; black arrows = blood vessels; red arrows = osteoclasts; white arrows = osteoblasts; gray arrows (B, D) = collagen fibers following an organized orientation; gray arrows (H) = collagen fibers interposed between the alveolar bone and root cementum; * (asterisk) = disconnected collagen fibers with presence of interstitial edema.
