# Supplementary figures and images for: Probiotic consumption reduces alveolar bone loss and kidney damage in pregnant rats with experimental periodontitis
Source: J Periodontol. 2025 Sep 26;97(4):732–46. doi: 10.1002/jper.11389 (PMC13169480; doi:10.1002/jper.11389)

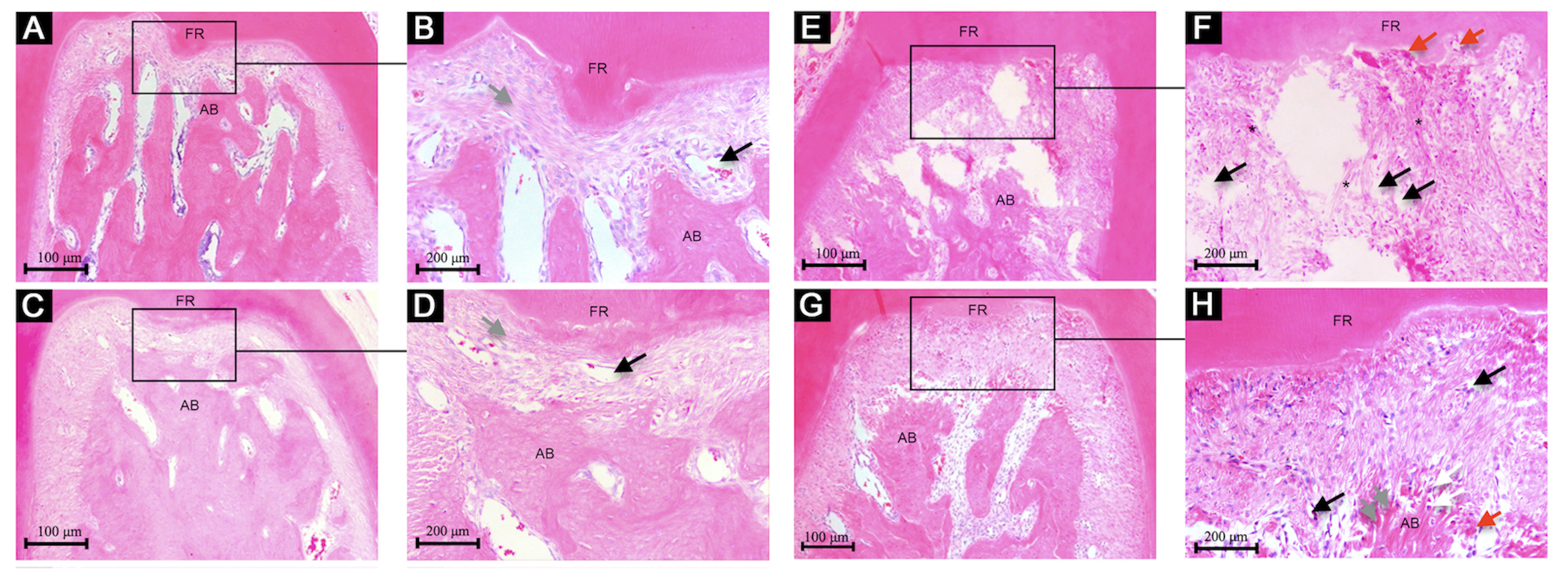

Supplement: Supplementary file 2 — Supporting Information [file JPER-97-732-s002.tiff]
